# Evaluation of Land Suitability Methods with Reference to Neglected and Underutilised Crop Species: A Scoping Review

**DOI:** 10.3390/land10020125

**Published:** 2021-01-28

**Authors:** Hillary Mugiyo, Vimbayi G.P. Chimonyo, Mbulisi Sibanda, Richard Kunz, Cecilia R. Masemola, Albert T. Modi, Tafadzwanashe Mabhaudhi

**Affiliations:** 1 Centre for Transformative Agricultural and Food Systems, School of Agricultural, Earth & Environmental Sciences, University of KwaZulu-Natal, P/Bag X01, Pietermaritzburg 3209, South Africa; 2 Department of Geography, Environmental Studies and Tourism, University of the Western Cape, Private Bag X17, Bellville 7535, South Africa; 3 Centre for Water Resources Research, School of Agricultural, Earth and Environmental Sciences, University of KwaZulu-Natal, P/Bag X01, Pietermaritzburg 3209, South Africa

**Keywords:** hybrid land evaluation systems, land management, machine learning, MCDM, NUS

## Abstract

In agriculture, land use and land classification address questions such as “where”, “why” and “when” a particular crop is grown within a particular agroecology. To date, there are several land suitability analysis (LSA) methods, but there is no consensus on the best method for crop suitability analysis. We conducted a scoping review to evaluate methodological strategies for LSA. Secondary to this, we assessed which of these would be suitable for neglected and underutilised crop species (NUS). The review classified LSA methods reported in articles as traditional (26.6%) and modern (63.4%). Modern approaches, including multi-criteria decision-making (MCDM) methods such as analytical hierarchy process (AHP) (14.9%) and fuzzy methods (12.9%); crop simulation models (9.9%) and machine learning related methods (25.7%) are gaining popularity over traditional methods. The MCDM methods, namely AHP and fuzzy, are commonly applied to LSA while crop models and machine learning related methods are gaining popularity. A total of 67 parameters from climatic, hydrology, soil, socio-economic and landscape properties are essential in LSA. Unavailability and the inclusion of categorical datasets from social sources is a challenge. Using big data and Internet of Things (IoT) improves the accuracy and reliability of LSA methods. The review expects to provide researchers and decision-makers with the most robust methods and standard parameters required in developing LSA for NUS. Qualitative and quantitative approaches must be integrated into unique hybrid land evaluation systems to improve LSA.

## 1 Introduction

The world’s population is projected to reach approximately 10.9 billion by 2021, and about two-thirds of the predicted growth in population between 2020 and 2050 will take place in Africa [1]. The population in sub-Saharan Africa (SSA) is growing at a rate of 2.7% a year and is expected to double by 2050 [2]. Farmers have a mandate to feed the growing population by sustainably increasing food production [3,4]. In the past, increasing food production was achieved by expanding the area under cultivation and increased contribution from breeding and agronomy, which resulted in improved output per unit area of land. However, there has been a significant decrease in arable land due to the expansion of urban areas, the spread of invasive alien species into farmlands, changing land potentials for agriculture due to climate change, land degradation and desertification [5]. While these challenges may differ in extent and magnitude, they have been severe in marginal communities that rely on agriculture as a livelihood strategy, have limited land tenure and lack the necessary resources to adapt to climate change. In response to these challenges, there is a need to redesign current agricultural landscapes, particularly those found in marginal communities, to improve crop production sustainably under the dwindling resource base and climate change [6].

Neglected and underutilised crop species are crops that have not been previously classified as major crops, are under-researched, occupy low levels of utilisation and are mainly confined to smallholder farming areas [7]. They are well known for tolerating adverse conditions such as those observed under climate variability and change, and marginal land [8,9]. They are an option for addressing dietary concerns that result in malnutrition [10]. Lack of attention from researchers has meant that their potential value is underestimated and underexploited; but, over the last decade, research on their production and use has gained considerable ground [11]. Despite this, the importance of NUS in rural food systems and information regarding their suitability across diverse agricultural landscapes remains mainly anecdotal with limited information detailing “where”, “why” and “when” they grow [12,13]. Such information is essential if NUS are to be incorporated into existing cropping systems, used to increase marginal landscapes’ productivity, and reclaim degraded agricultural land.

Cropland identification and classification exercises address questions such as “where”, “why” and “when” a particular crop is grown for a specific area [14,15]. To date, there are many different land suitability analysis (LSA) methods [16]; this suggests there is no universal and exhaustive process. Land suitability analysis is a process applied to determine a specific area’s suitability for considered use; it reveals the suitability of a site regarding its intrinsic characteristics (suitable or unsuitable) [17]. After that, land suitability mapping can be used to address the questions “where” in terms of land and resource use; hence establishing conditions favourable for sustainable production of a particular crop [18]. Due to the large number of factors considered during LSA, the process is often identified as Multi-Criteria Evaluation (MCE) [19]. Cropland identification and classification exercises address questions such as “where” and “why” a particular crop is grown for a specific area [14,15]. To date, there are many different land suitability analysis (LSA) methods [16]; this suggests there is no universal and exhaustive process.

Geographical Information Systems (GIS) have become central to LSA, as it allows the investigation of multiple geospatial data [20,21]. The integration of remote sensing (RS), machine learning tools and techniques, use of big data, Internet of Things (IoT), blockchain and cloud computing to form hybrid land evaluation systems can improve the accuracy and reliability of land suitability methods [22]. In hybrid land evaluation systems, the linkages between two types of models simulate both the qualitative reasoning functions and the quantitative modelling part [23]. In recent years, mechanistic crop simulation models have proven useful in optimising and developing hybrid land evaluation systems [24,25]. Nevertheless, LSA has often focused on commercially essential field crops and methods for analysing suitability, and their application within NUS is yet to be established. Due to the limited scientific knowledge of NUS, it is imperative to develop appropriate methods and tools that can be used.

Decision-makers require knowledge concerning NUS’s current spatial occurrence and the interaction of biophysical and socio-economic factors to detect both threatened areas and potential growing zones, especially in semi-arid and arid regions [26]. Mapping NUS’s potential spatial distribution is a transformative agenda to achieving food and nutrition security goals in marginal environments [26]. Given the need to mainstream NUS into existing agricultural landscapes, there is a need to identify reliable land suitability approaches and methods. Therefore, the review synthesises the existing techniques, methods and tools that can be used to develop land suitability maps that can be applied to NUS. This will be done by addressing the following research questions: which methods have been used to assess land suitability for crop production, and which parameters have been used in developing land suitability? Perspectives for future research will be provided that recognise the land cover aspect without further characterisation of land use in terms of NUS suitability and management interventions. This review also identifies parameters and common LSA methods that can help researchers, practitioners and policymakers to develop guidelines on the successful crop suitability mapping process for improved crop productivity. Therefore, the optimum method for land suitability should consider the cost of tools, the complexity of the procedure and benefits in handling a specific land evaluation.

## 2 Methodology

### Literature Search

A scoping review approach was used to acquire and synthesise information on land suitability for crops. Previously, there were 11 review studies related to land evaluation on agriculture and environmental studies; however, few of them focused on land suitability analysis for crops [15,19,27]. In terms of literature, the review sourced information from 1993 to 2019 using the Preferred Reporting Items for Systematic Reviews and Meta-Analyses [28] (Supplementary Materials, Figure S1). Literature was sourced from Scopus and Web of Science using a Boolean search approach. The following search syntax was used ((“land suitability” OR “land suitability analysis” OR “land evaluation methods” OR “species distribution models” OR “habitat suitability” OR “bio-climatic models”) AND (crop* OR plant* OR yield OR agriculture). The search was limit to titles, abstract and keywords. This search identified 786 and 737 articles in Scopus and Web of Science, respectively. Identified articles were exported to Mendeley^®^ as BibTex files and duplicates were removed, leaving 876 articles. Articles assessing land suitability of a range of crops including annual food crops, shrubs and trees or non-food crops or animals and invertebrates were retained for further analysis. Following the screening, 131 abstracts were identified, and of these, 101 full-length papers were downloaded and used in the analysis. Where available, full-length articles downloaded and research study details were extracted, such as the country where the study was carried out, the study’s objective, methods or model used, crop(s) studied, whether it was an NUS (Yes/No) as presented by the priority list for SSA (see Williams and Haq [29] and Mabhaudhi et al. [8]) for full list), and the thematic factors used in assessing suitability. We developed a Microsoft Excel spreadsheet to enter and later quantitatively assess the extracted data. We assumed no selection bias as the literature search and curatorship were done by two independent researchers.

## 3 Results

### 3.1 Results of Literature Search

**Figure 1:**
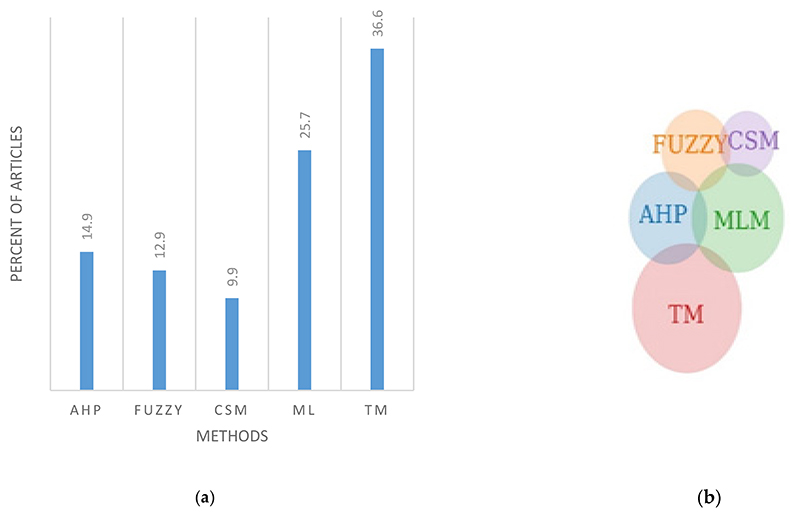
(**a**) The percentage distribution of land suitability methods published from 1993 to 2019. (Analytical hierarchy process (AHP), Crop Simulation Models (CSM), Machine learning method (ML/MLM), traditional method (TM)). (**b**) The hybrid land evaluation systems, the combination was selected from land suitability methods published from 1993 to 2019. (Analytical hierarchy process (AHP), crop simulation models (CSM), machine learning method (MLM), traditional method (TM)).

Following the systematic search, 101 papers were selected for further analysis (Supplementary information). From the articles reviewed, only five crops were regarded as NUS (sorghum [30–32], cassava [33,34], cowpea and pearl millet [35], and foxtail millet [36]) have been assessed and these were across 23 articles (Tables S2 and S3; Supplementary information). The majority of crop species were cereals, namely maize, rice and wheat. The legume soybean and the tuber potato have also been assessed extensively (Tables S1 and S2; Supplementary information). The highest number was from Iran and a few for Africa. From the identified literature used in this review, 36.6% used empirically called traditional methods in Fig. 1a and 1b. derived methods, 25.7% used machine learning related methods, and 14.9 and 12.9% used AHP and fuzzy approaches, respectively. It was interesting to note that 9.9% of the articles used crop simulation models (Figure 1a). The hybrid methods use more than one technique to assess suitability, were the ones that integrated AHP with Machine Learning methods (MLM) (e.g., Habibe et al. [37] (Table 1). The least common hybrid method was that between Fuzzy and Crop Simulation Models (CSM) methods. Based on the reviewed literature MLM were the most versatile and could be integrated with other LSA methods (Figure 1b). There were no articles that showcased the integration of AHP and CSM or TM with either CSM or FUZZY from the identified literature. The distribution of methods discussed is indicated in Figure 1, and a full list of journals is provided in (Supplementary Tables S1–S6). Across the identified articles, the terms land capability and land suitability were often used interchangeably, although they refer to different types of appraisals in the stricter sense. According to Neitsch et al. [38], land capability is the land’s inherent capacity to perform at a given level for general use. Rabia et al. [37] defined land capability as a land classification primarily based on degradation hazard. According to Teixeira et al. [39], the term ‘land capability’ is based on assessing soil conditions that support cultivated crops. Examples of such systems include the Canada Land Inventory and the USDA land classification system [39]. Since the issue that we are trying to address with NUS goes beyond the bio-physical attributes within agriculture and speaks to socio-ecological characteristics of an area, land suitability is most appropriate. Table 1 provides an overview of hybrid land evaluation systems used in cropland suitability assessments.

### 3.2 Approaches to Land Suitability Analysis

Land suitability analysis depends on several factors: data availability (quality and quantity), expert skills and the end-use of suitability assessments. Therefore, having a universal technique is not always feasible. In NUS’s context, they are a vital source of agro-biodiversity, are socially and culturally significant for marginalised communities and can address pertinent challenges such as building resilience to climate change [8]. The assessed literature includes a wide range of approaches, differing in level of complexity and data requirement. According to Akpoti et al. [19], these LSA methods can be categorised as traditional or modern methods dealt with in Section 3.2.1.

#### 3.2.1 Traditional Land Suitability Methods

In traditional LSA methods, biophysical characteristics are used to assess crop options using qualitative, quantitative and parametric methods (Supplementary Table S1). According to Manna et al. [46] and Akpoti et al. [19], qualitative approaches assess land potential in terms of the degree of suitability, such as highly, moderately, or not suitable [53]. On the other hand, quantitative assessment methods give numeric indicators and use mathematical models to describe physical conditions of geo-biophysical scenarios [54]. Qualitative approaches evaluate land on a broader scale depending mostly on land uses while the quantitative approach comprises more detailed technical procedures [19,55–57]. Within these procedures, arithmetical or parametric methods consisting of statistical analysis are applied [55,56]. The difference between the two approaches lies in the technical procedures adopted for land evaluation [57,58]. In the context of promoting NUS in the marginal cropping system, LSA methods selected to delineate homogenous zones should accommodate minimum multidisciplinary data to map land units with homogenous zones. The low requirement is because NUS have poorly developed knowledge systems and lack empirical data on how they can be cultivated. In this regard, parametric methods, that is, the integration of both qualitative and quantitative approaches to form hybrid land evaluation systems have been used to improve the accuracy, reliability and applicability of land suitability analyses to real-world challenges [59].

Parametric methods are derived from the numerical inferred effects of various land characteristics on a land use system [16]. These methods allocate a numerical value on the most significant land characteristics. They account for interactions between factors expressed through a simple multiplication or addition of single-factor indices [60]. The main weakness of parametric methods is that the scores can be either very small or very large, which affects the overall suitability [61]. Another bottleneck of the parametric method is the absence of any uncertainty or vagueness associated with factors determining land use suitability for crops [42]. Then again, within the context of promoting NUS, a socially and economically relevant subset of agrobiodiversity, it is vital to consider using a hybrid land evaluation systems to capture both the qualitative and quantitative properties in NUS.

Several methods that have been coined “traditional” but are still widely used and include Boolean logic [62], weighted linear combination (WLC) [63], weighted overlay (WO) [64], storie and square root [65], multiple linear regression models [66] and multivariate statistics [19] (Table 2). Among the traditional methods, categorical data is limited except on the WLC and qualitative approach (Table 2) [67]. According to the literature, the Food and Agriculture Organisation approach has been used as a major LSA framework for assessing crop suitability [68,69]. Across most of the identified traditional methods, socio-economic data is minimal, yet socio-economic data is critical when conducting any assessments for crops such as NUS. Also, Hopkins [58] pointed out limitations associated with using ordinal, linear combination methods, which can be addressed using a combination of non-linear methods. Manna et al. [46] concluded that changing land use and management practices must be based on land evaluation results on suitability and vulnerability, thus transcending the reductionistic approaches of qualitative and quantitative methods. Table 2 provides an overview of selected traditional methods used in land suitability assessments.

##### The FAO Approach

The FAO Land Evaluation Framework was published in 1976 [68,69]. The Food and Agriculture Organisation (FAO) approach evaluates the suitability of land for specific land use rather than general land use, of which the latter often denotes land capability. The FAO approach seeks to match land utilisation types with the land use requirements across land units [53]. This approach requires a description of the land in terms of its characteristics to the intended use. The method differentiates between land suitable for crops (S) or not suitable for crops (N). At the same time, classes show the degree of land suitability, such as (S1) highly suitable, (S2) moderately suitable, (S3) marginally not suitable, (N1) currently not suitable and (N2) permanently not suitable [69].

It uses a Boolean mapping approach that ignores the continuous soil variation, and possible uncertainties in measurement [62]. As such, the implicit assumption in Boolean approaches is the absence of any uncertainty or vagueness associated with the land suitability analysis, measurement, imprecision, and specified concepts [42]. These assumptions may be invalid in smallholder farming systems where many could be located on similar land classes but are highly variable in the social and economic landscape and across farming activities. The FAO approach can also result in areas with variation in soil texture, depth, pH, and landscape being excluded from the set of suitable land because they fail to match strictly defined requirements [70]. Then again, the framework tends to be a top-down approach, which ignores the social constructs of the land being evaluated. In reality, NUS are suitable in marginal areas with high climate, soil and landscape variation; there is a need for methods that capture uncertainties and data variation. To address some of these challenges, one of the most significant developments in the FAO approach has been the advent of an affordable computer-based (vs mainframe) geographic information systems (GIS) and machine learning skills. The integration of FAO methods and modern methods ensures that an objective LSA for NUS can be carried out.

Geographic Information System tools and machine learning skills ease the storage and analysis of a wide range of spatial data [71]. Despite the significant development of modern LSA methods such as crop simulation and machine learning tools, the FAO conceptual land evaluation framework gives the basic guidelines in agriculture to carry out a land evaluation process [72]. Land suitability from the FAO method does not necessarily identify a single index of use as best on each land unit; the results become qualitative [73]. Multi-criteria decision-making (MCDM) methodologies, which fall under modern methods, have been proposed for overcoming problems related to vagueness in definition and other uncertainties, especially in the context of NUS suitability analysis [19].

#### 3.3.2 Modern Land Suitability Approaches

Akpoti et al. [19] classified modern LSA methods as those combining GIS and machine learning algorithms (Table 3). They are termed modern land suitability approaches because they integrate several variables to map areas with homogenous characteristics. The modern LSA methods are populated by more complex and often time-consuming and more dynamic algorithms [71]. The modern methods are often grouped into three major categories: (i) computer-assisted overlay mapping; (ii) soft computing or geo-computation, also known as artificial intelligence (AI); (iii) multi-criteria evaluation (MCE) or multi-criteria decision-making (MCDM) [16,17,19].

The use of more than one MCDM method to form a hybrid land evaluation system in LSA allows approximate representations of vague, incomplete and uncertain information because land suitability will be defined as continuous classes, rather than “true” or “false” as in the Boolean model [16]. Use of MCDM methodologies in NUS can provide better land suitability than Boolean approaches because they can accommodate attribute values and properties that are close to category boundaries. Table 3 provides an overview of selected modern land suitability methods.

##### Multi-Criteria Decision Analysis

**Figure 2:**
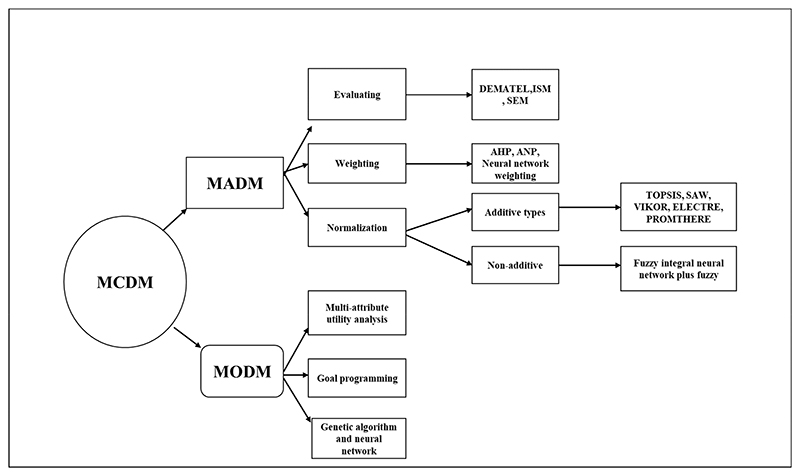
MCDM classification split between MADM and MODM.

Due to the many attributes and criteria involved in decision-making, land suitability evaluation has been identified as a multi-criteria evaluation problem. To address these challenges, Multi-criteria Decision Analysis (MCDA) was developed in the 1960s to assist decision-makers in incorporating many options, into a potential or retrospective framework [74]. Multi-criteria decision analysis involves input data (from socio-economic, bio-physical and geopolitical domains), the decision maker’s preferences, and manipulation of both using specified decision rules [75]. Using GIS tools, the information is combined to form a single index of evaluation [74]. Geographic information system tools are best suited for handling a wide range of criteria data with different spatial and temporal scales from different sources for a time-efficient and cost-effective analysis [75]. Multi-criteria Decision Analysis approaches that are GIS-based are useful because various production variables can be evaluated, and each weighted according to their relative importance on the optimal growth conditions for crops [54]. Then again, its use involves developing an optimisation suitability index derived from heterogeneous data [76,77]. This is a challenge because weights given to parameters depends on subjectivity. Malczewski [55] and Leake and Malczewski [77] classified decision support models into Multi-objective decision making (MODM) and multi-attribute decision making (MADM) (Figure 2). In MADM, methods are considered data-oriented. The goal is to design the best alternative [77,78]. The MODM uses a series of mathematical models where alternative decisions are not predetermined, but instead, are a set of objective functions to be optimised [78]. Multi-attribute decision-making methods can be classified as:

- Weighting methods (linear additive model, AHP and the multi-attribute utility theory).
- Multiple objective programming (Multi-objective linear programming).
- Outranking approaches (ELECTRE, PROMETHERE).

Spatial MCDM has also become one of the most useful methods for land use and environmental planning as well as water and agricultural management. The use of free spatial data will go a long way to solve land suitability issues, especially in areas where input data is not readily available. Spatial MCDM is more complex and challenging than conventional MCDM, as many factors, with strong correlations between them, are needed [79]. In the context of fuzzy set theory (FST), which expresses uncertainties in human opinions, can be successfully used together with MCDM methods to get more sensitive, concrete, and realistic results [20,80–82]. Also, Kaya [83] indicated that AHP, when used as an individual tool or integrated with another MCDM method, is the most applied and preferred MCDM method, since it can handle a large degree of uncertainty in linguistic terms during decision ranking. Such integration is important when mapping the suitability of NUS because it considers many factors affecting crop production.

##### Analytical Hierarchy Process

Analytic Hierarchy Process (AHP) is a tool for dealing with complex decision making [78,84]. In agriculture, AHP is the most widely accepted method and is considered the most reliable MCDM method [85]. It can be used as a consensus-building tool in situations involving a committee/group decision making [78]. The AHP helps capture both subjective and objective aspects of a decision by reducing the complexity of pairwise comparisons and then synthesises the results to a single index [22,86]. The AHP considers a set of evaluation criteria and alternative options from which the best decision is made. It generates a weight for each criterion according to the decision maker’s pairwise comparisons [87]. The higher the weight, the more critical the corresponding criterion [20]. Next, for a fixed criterion, the AHP assigns a score to each option according to the decision maker’s pairwise comparisons based on that criterion [20]. The higher the score, the better the performance of the option concerning the considered criterion. Finally, the AHP combines the criteria weights and the option scores, thus determining a global score for each option, and a consequent ranking. A given option’s score is a weighted sum of the scores it obtained for all the criteria [20]. Although the AHP can solve complex spatial scenarios, it has some limitations in consistency and is subjective [88]. In AHP, weights given to inputs depend on a scientist’s expertise, though it can be improved by:

(a) “Deriving a pairwise matrix based on a scientific objective in a non-scarce data situation [89]”.
(b) Estimating relative importance of factors individually and based more on scientists’ opinion through a questionnaire or focus group discussions with key informants like the Ministry of Agriculture, the farmers who grow the crops, agronomists and extension officers.
(c) Giving attention to an upper limit, where the upper limit is a consistency ratio (CR) that must be less than 0.1 for a pairwise matrix judgment to be accepted [90]. To minimise the interrelationship among various factors included in the AHP approach, a data reduction method such as principal component analysis (PCA) can combine factors as fewer new variables [74].
(d) The process is based on three principles: decomposition, comparative judgment and synthesis of priorities. Manipulating three principles, for example, synthesis of priorities can be used to evaluate land use opportunity costs, especially when NUS production can complement major crops in semi-arid areas to improve food security.

The AHP uses a 9-point scale measurement (1 = equal importance, 3 = moderate importance of one over another, 5 = strong or essential importance, 7 = very strong or demonstrated importance, 9 = extreme importance, and 2, 4, 6, 8 = intermediate values) to express individual preferences or judgments [91]. It is important to note that, since some of the criteria could be contrasting, it is not true in general that the best option is the one that optimises every single criterion, rather the one which achieves the most suitable trade-off among the different criteria. The weighting of parameters for AHP suitability can be estimated using a geometric mean method [92]. Though AHP can be used as a decision tool, it can be combined with other MDCM methods like fuzzy logic to create a unique hybrid land evaluation system [93]. The procedure considers the spatial planning decision context, identifying and arranging the criteria into different groups [20,94]. To date, in crop production, several (20.3%) articles used the AHP method to generate land suitability mainly for major crops like maize and potatoes [12] (see Supplementary Table S2). Use of AHP in NUS’s land suitability will help develop a quantitative index from heterogeneous data to indicate suitable areas.

##### Fuzzy Logic Technique

Fuzzification is the process by which crisp attribute values are mapped into a common suitability scale by using membership functions [95]. The attributes measured using different scales are converted into a standard range called fuzzy sets [96]. Since the approach is based on “degrees of truth”, the technique is useful in a scenario where data for classification is limited. It cannot be used where actual boundaries are needed [97].

The fuzzy method is common in LSA because it can characterise vague and uncertain objects in classification since it does not have any definite boundaries [98]. Fuzzy logic requires fewer data to run the model; therefore, it can be manipulated to map NUS in agroecologies where there is limited information about their production [99]. Also, fuzzy logic techniques can be used where the ethnobotany of NUS is poorly documented and patchy. Most of the available social ecology datasets are categorical, and they require flexible models such as fuzzy logic [100]. The method’s flexibility allows it to be combined with other methods making it suitable to map complex systems like those where NUS might be found suitable. Feng et al. [101] assessed the suitability of switchgrass using a fuzzy logic technique, one-step-at-a-time method, and weighted linear combination. It is also possible to use the Law of the Minimum to provide a consistent framework to assess climate suitability of crops using a fuzzy logic model. The Law of the Minimum is the outcome of fuzzy intersection using the minimum t-norm between those propositions [102]. However, NUS are mostly grown in remote rural areas where production information is scarce and not documented. Therefore, the results from fuzzy indices cannot be used where precision agriculture is required to achieve sustainable intensification of NUS [9].

##### Crop Simulation Models

Crop simulation models (CSM) are considered as one of the most reliable ways to measure land suitability in the context of specific crop requirements within a defined cropping system. A CSM is a mathematical model that describes crop growth and development as a function of weather conditions, soil conditions, and crop management. According to ecological drivers, they simulate biological processes and account for the interactions of weather, soils, and management factors [31]. Many of the popular models (e.g., DSSAT, CropSyst [103,104], CROPWAT [105,106], CROPGRO [107], and APSIM [108].) are process-based; they simulate critical physiological processes such as crop development, net carbon assimilation, biomass partitioning, crop water relations, and grain/fruit growth using point input data [31]. Therefore, such models are useful decision-making support tools in agriculture and land use.

Several crop models have been used to evaluate crop suitability at different scales [80]. For instance, the EcoCrop model [109], is a simple empirical model intended for suitability assessments of crop species for which there is not enough agronomic data to run more complex (process-based) models. This model has been used on sorghum [31] and various food crop species that included NUS [110]. The MicroLEISDSS model has evolved significantly towards a user-friendly agro-ecological system for sustainable land management; it has been used to predict agricultural land suitability [111]. Also, CSM can be used to validate suitability indices from other LSA methods. For example, Estes et al. [44] used a mechanistic crop growth model (DSSAT) to validate a maize suitability index for South Africa that was derived from using MaxEnt in South Africa. Hence, CSMs can be used as a scientific method to validate land suitability indices derived from species distribution models [111].

Then again, CSMs often rely on massive datasets with long time-series and highresolution data, often not available at national or continental scales. Following the increase in free online remote sensing datasets (big data), CSMs are gaining popularity because they can process a high volume of data [71]. Another important criterion to consider is whether the model can be run in “batch mode” or gridded mode. Kunz et al. [112] noted that considerable effort was spent on automating the standalone version of AquaCrop to enable the model to run non-stop at a regional and national level in South Africa. They noted that over 5000 lines of computer code were written to facilitate this process. Similarly, the APSIM model can also be run in gridded form from a command-line prompt without the user’s need to interact with the model. Hence, model runs can also be automated as was done for AquaCrop. To date, 9.9% of 101 articles in land suitability mapping used CSMs (Supplementary Table S3). Despite efforts to use CSMs in NUS research to develop crop production guidelines [113,114], the approach depends on the availability of input data like climate data, which may be unavailable in some areas. Furthermore, the use of CSMs requires expert skills.

##### Machine Learning-Related Methods

Artificial intelligence through machine learning algorithms is gaining popularity in land suitability analysis [55,115]. The technique can handle large time series and categorical datasets for land evaluation obtained from remote sensing, climate models, and direct field data collections. The ability to automate land classification through machine learning algorithms has emerged as a critical modelling tool in land suitability analysis [116]. The machine learning method (MLM) can be defined as a data analysis method that automates multivariate data by using statistical analysis and validated approaches [40]. Commonly used methods are Artificial Neural Networks (ANNs), Logistic Regression, Regression tree, Cellula automata (CA), Markov chain, fuzzy rule-based systems, goal programming, species distribution models like MaxEnt, and Global Environmental Stratification Strata (Supplementary Table S4).

Machine learning algorithms have several advantages. For example, no human intervention needed (automation), easy identification of trends and patterns, and the ability to handle multi-dimensional and multi-variety data required in NUS land suitability analyses. However, MLMs are not perfect as they require massive data sets to train. These should be inclusive/unbiased, which is a significant limitation in NUS production in marginal areas. Elith et al. [117] noted that high collinearity is less of a problem for MLMs than statistical methods. However, we caution that this is only true if the presences’ predictive accuracy is the study goal. Coding ML algorithms require programming skills, which are still a challenge in most African regions. Therefore, the use of window-based MLMs such as MaxEnt can help to map NUS. The MaxEnt software package can accommodate non-parametric and parametric datasets; however, it uses the machine learning approach by default [118,119].

##### Species Distribution Models

Understanding species geographic range has become more critical with concerns over climatic variability and change and the need to fit adaptable crops within a defined construct. In this case, fitting of NUS into marginal production systems. Species distribution models (SDMs) are used to simulate species’ suitability in ecology [24]. They can estimate changes in habitat suitability and identify conservation priorities [120]. These models are used to match crop phenology and bio-physiological and then calculate the suitable area for a crop [121,122]. They are also used in climate change studies to quantify species-environment relationships to inform management, assess assemblage changes under different land-use patterns, predict responses to future climate or restoration scenarios, aquatic mapping biodiversity, and identify species conservation priorities [121,122]. Species distribution models have been used to predict the potential growing areas for potatoes in Australia [123]. This is done by identifying environmental determinants of species suitability by assessing the relative importance of predictor variables (e.g., climate) and examining the crop response curves in partial regions of selected predictor variables [124]. Species distribution models could be used to examine climatic suitability of a crop [115].

Several machine learning-based SDMs are widely used to generate bioclimatic models for predicting the geographic range of organisms as a function of climate [124]. However, the success of machine learning-based approaches depends on its ability to distinguish heterogeneous zones. Therefore, SDMs require evaluation to measure sensitivity and accuracy through confusion matrices [125]. The process of evaluating the suitability for a specific purpose requires a comprehensive analysis of both natural and socio-economic factors [36,55]. Despite their applicability, SDMs also require many input variables and need to be trained with presence species data to predict crop suitability zones [126,127]. There is a problem of overfitting if more variables are used in land evaluation process [128].

The different use of SDM and several studies indicated that climate changes have already affected species’ geographical distributions [129]. Nevertheless, SDMs have certain advantages and disadvantages as per review by Austin [129]. They offer a tool for undertaking relatively rapid analysis for numerous individual species and identify critical relationships between a species and its distribution governing factors. However, the drawback with most land suitability assessment studies using the SDMs is that they tend to be general and assume a linear relationship. However, in reality, an environment’s suitability to NUS is a function of complex interactions between various factors operating at different scales and magnitude [130].

### 3.3 Combining Geographic Information System, Remote Sensing and Other Artificial Intelligence Tools

A land suitability analysis should identify innovative ways to derive maximum value from the possible integration of GIS with, big data, and IoT technologies. The GIS and other artificial intelligence tools can handle the volume of data with different structures, especially the socio-economic data, which is usually in categorical form [25]. The use of wireless sensor networks with IoT based applications should be used to measure LULC changes. It is understood that the IoT applications in crop suitability will empower the majority of the NUS-related industries to extend their value chains to cater to their stakeholders resulting in increased profitability [131]. The IoT is one of the up-and-coming technologies which provides many techniques for modernising land suitability methods. The IoT supports interoperability among various connected devices and helps obtain the much needed near real-time information in land suitability [22]. Drones use automated control systems and can provide the necessary geospatial data, thus reducing the complexities involved in capturing field data [132].

Future research studies should focus on developing intelligent decision support systems for land suitability analysis and a web-based spatial decision support system [133]. Future studies should integrate GIS, remotely sensed data, computer modelling, and MCDM approaches within a hybrid land evaluation system to deliver better insights into land suitability to improve the strategic, tactical, and an operational level of decision making [134]. [132] suggested using a windows-based GIS application with an artificial neural network (ANN) to delineate land suitable for crops. Similar approaches will need to be adopted in future studies in NUS with a specific focus on land suitability.

Remotely sensed imagery could be integrated within a GIS. For land suitability analysis, remote sensing plays a vital role both at regional and local levels. It also offers an efficient and reliable method of mapping agricultural lands. Spatial crop suitability use information is one of the key input parameters for agroecosystem modelling [135]. In RS, big data challenges are not limited to the analysis of high volumes of data, but also involves big data acquisition, storage, management and analysis. Tripicchio et al. [132] proposed that studies should focus on designing high-performance systems for easy access to distributed data by different users. Systems that use cloud computing are useful in overlaying multiple data from different sources. The major challenge in remotely sensed data is its ownership and connectivity between the different stakeholders in agriculture. Big data analysis requires modern computing and analytical methods to analyse the unevenly distributed data originating in near real-time from different locations. Therefore, future studies should focus on developing new algorithms that can be used to develop land suitability maps that are not static but rather dynamic to factor in climate change and climate variability effects.

### 3.4 Hybrid Land Evaluation Systems

In recent years, NUS studies have gained momentum with a lingering question on how and where they fit in the current agricultural landscape. Land suitability analysis for agriculture is an important technique in deciding future agricultural cropping patterns, planning and activities. Consequently, land suitability is decided on the merits of each land unit’s bio-physical and socio-economic properties. Most, if not all, methods reviewed in this study can be used to assess NUS suitability in agricultural landscapes; however, each method carries some limitations. For instance, in AHP, the consistency of original datasets, biased weighting and selection criteria may result in uncertainties is final decisions. Akpoti et al. [19] indicated that the fuzzy logic approach’s main limitation is the lack of a definite method for determining the membership function, which is often based on expert opinion. The integration of RS-GIS, fuzzy-logic and multi-criteria evaluation using the analytical hierarchy process (AHP) could provide a superior database and guide map for decision-makers considering cropland substitution to achieve better agricultural production. It was interesting to note that 14.8% of the articles used HLES (Table 1). The review identified that there is no single method that is supreme. LSA’s application depends on data availability, type of data, expertise, available software, and objective of the exercise [19,72]. Although we recommend HLES, the hybrid method did not come out as the panacea of methods but to acknowledge that a lot of research is gravitating towards them, especially for planning and monitoring purposes and climate change-related issues.

In land evaluation hybrid systems, the linking of more than two types of models is gaining momentum in LSA [60]. The HLES can combine traditional land evaluation systems and crop models to give land suitability for crops and formulate strategies to promote NUS in marginal lands [136]. Following attempts to combine land evaluation methods with crop modelling, newly developed hybrid methods have captured and handled multidisciplinary data sets. However, this is often not possible due to lack of data, the most important being climatic data, phenological information, recorded yields, primary social-economic data such as costs, availability of markets, management and agricultural inputs [8,11]. For example, Bonfante et al. [136] developed and tested a hybrid land assessment methodology to demonstrate the impact of climate change on Italy’s maize varieties. Applying these methodologies to minor crops and their landraces will require some compromise in defining unknown crop growth parameters [137]. Jahanshiri et al. [137] noted that assessing the potential of land for crop diversification involving NUS at a specific location requires a practical approach that takes advantage of available data and knowledge. Hence, GIS and machine learning skills have seen a drastic evolution from traditional practices involving land use planning to new land evaluation methods. Big data, cloud computing, Internet of Things (IoT) and other technological advancements improve the accuracy and reliability of land suitability methods [71]. The availability of accountable and reliable free online data is expected to play a significant role in shaping land use planning because local datasets are not readily available in many cases.

### 3.5 Factors Considered in Crop Suitability Mapping

The mapping and the accuracy of land use systems and their associated characteristics depend on the scale and availability of data at an acceptable resolution. The process of evaluating land suitability for a specific purpose requires a comprehensive analysis of natural factors and socio-economic factors which influence the land [36,55]. The elements used can be divided into high and lower factors based on experts’ opinion weightings [138]. High-level factors are natural or biophysical factors that directly affect crop growth, such as rainfall, temperature, and soil fertility. Lower-level factors are social and economic factors that not physically affect crop growth but influence the land use degree of appropriateness to a specific purpose. The interactions, dependencies and feedback between higher and lower-level elements form a multi-criteria land evaluation approach for a sustainable NUS production [139]. Multidisciplinary factors were ranked to show the most commonly used factors (Supplementary Table S6). The factors were grouped into climatic indicators, hydrology, soil and landscape attributes, land use land cover, and socio-economic and technical indicators. At the current time, many different sources of climate data are freely available on the web, like WorldClim and Environmental Raster for Ecology Modelling [119]. The description and importance of each factor are beyond the scope of this review; however, for a detailed description, readers may refer to Akpoti et al. [19] The mapping and the accuracy of land-use systems and their associated characteristics depend on the scale and availability of data at the accepted resolution. The process of evaluating the suitability for a specific purpose requires a comprehensive analysis of natural factors and the socio-economic factors which influence the land [58,126]. The elements used can be divided into high and lower factors based on experts’ opinion weights [140]. High-level factors are natural or biophysical factors that directly affect crops’ growth, such as rainfall, temperature and soil fertility. The lower-level factors are social and economical and do not physically affect crop growth but influence land use degree of appropriateness to a purpose. The interactions, dependencies and feedback between higher and lower-level elements form a multi-criteria land evaluation approach for a sustainable NUS production. Multidisciplinary factors were ranked to show the most commonly used factors (Supplementary Table S6). The factors were grouped into climatic indicators, hydrology, soil and landscape attributes, land use land cover and socio-economic and technical indicators. At the current time, many different sources of climate data are freely available on the web, like WorldClim and Environmental Raster for Ecology Modelling [119]. The description and importance of each factor is beyond the scope of this review; however, for detailed description readers may refer to Akpoti et al. [19]

Understanding land use and land cover (LULC) change patterns is vital for crop suitability analysis and efficient environmental management, including effective water management practice [71]. To fit NUS in a farming system, updated LULC maps must be used to understand the proportion of land use pattern to guide planners to make more informed decisions and achieve a balance between urban growth and preserving the natural environment. Of all possible methods, that can delineate NUS, only 29% used LULC (Supplementary Table S6. Understanding land use and land cover (LULC) change patterns is vital for crop suitability analysis and efficient environmental management, including effective water management practice [72]. To fit NUS in a farming system, updated LULC maps must be developed to understand the proportion of land use is essential for the development of control measures, guide planners in making more informed decisions and achieve a balance between urban growth and preserving the natural environment. In all possible methods, which can delineate NUS, only 29% used LULC (Supplementary Table S6).

## 4 Discussion and Way Forward

The use of NUS to address food and nutrition insecurity, unemployment and rural development has been advocated for; however, their production continues to be disconnected from the current agricultural production system. It is widely believed that NUS offer more options for building temporal and spatial diversity into cropping systems [113,141]. However, this information is largely anecdotal. The paradox of being widely adapted to diverse agroecologies while having little to no information detailing land suitability makes it challenging for policymakers to mainstream NUS into current agricultural programs. Many studies have used MCDM techniques for analysing the complexities involved in land capability and suitability evaluation in crop production. However, all land suitability analysis methods are imperfect and require careful testing and evaluation before application [17,19,27]. To improve land use planning and give a real picture of land use, especially in smallholder farming systems, socio-economic factors should be included, where available [71,139]. Integration of quantitative simulation modelling and qualitative land evaluation techniques leads to excellent scientific and practical results which gradually improve the accuracy and the applicability of the models [23]. Finally, the practical automated application of land evaluation systems is described as a land-use decision support tool that uses information technologies to link integrated databases and various models [40]. Therefore, future research studies should consider using a broader range of attributes, including socioeconomic factors, of a hybrid land evaluation system for NUS LSA.

The development of artificial intelligence (AI) in LSA accommodate more multidisciplinary datasets [140]. It includes programming techniques of calculation that may help describe complex inference systems and decision-making [141]. The use of MLMs gained popularity in recent years [142]. With the development of technologies (GIS and machine learning), it is imperative to use MCDM. There is considerable potential for integrating big GIS analytics (BGA) in agriculture with other technologies such as LiDAR to improve land suitability mapping. The integration of analytical techniques (hybrid methods) will improve land suitability mapping, resulting in future climate-related risks based on past and current trends. The spatial analysis development showed that artificial intelligence (AI) offers new hybrid land evaluation systems and planning [143,144]. It includes programming techniques of calculation that may help describe complex inference systems and decision-making [145]. The ability of GA to GIS-based land-use suitability analysis has gained popularity in recent years [146]. With the development of technologies (GIS and machine learning) is very important to use MCDM. There is considerable potential for integrating big GIS analytics (BGA) in agriculture with other technologies such as LiDAR to improve land suitability mapping. The integration of analytical techniques (hybrid methods) will improve land suitability mapping, resulting in future climate-related risks based on past and current trends.

Future studies should focus on using new predictive tools in forecasting. It is observed that the majority of the studies in resource allocation utilised primitive GIS techniques. In resource allocation, GIS is a powerful tool for spatial analysis. As land resources are being depleted drastically, effective land use planning needs to be done to identify new crop production areas. However, the studies by Rey et al. [147] and Singh et al. [148] have used advanced geomatic tools for improving resource allocation. Models for simulating crop production and distribution are gaining attention from the research community [116].

Additional studies in resource allocation using geomatics are required in different regions. GIS-based “Environmental Policy Integrated Climate” (GEPIC) model, which is used for predicting crop production levels incorporating the near-real-time changes in crop environment, can be integrated with other techniques for improved decision-making [148]. Future studies should combine the GEPIC model with other methods to form a hybrid land evaluation system to assess the spatial distribution and stimulate crops’ production. Models for simulating crop production and distribution are gaining attention from the research community [116]. The use of advanced simulation software helps to remove the redundancy of the other processes and increase accuracy. Hence, researchers should focus on carrying out studies involving new and upgraded GIS software. Aerial vehicles (UAVs) may increase outreach to enhance resource allocation effectiveness [134]. Modelling techniques can be used for practical impact assessment of resources. This is evidenced by the study carried out by Estes et al. [45]. Future studies can focus on the use of mathematical tools for enhanced output.

## 5 Recommendations

- To efficiently identify homogenous zones, especially for NUS, hybrid methods are needed—approaches that combine traditional and modern methods (e.g., MCDM, CSM and MLMs). Suitable hybrid land evaluation systems may be useful in handling complexities such as the extreme variability, intermittence and socio-economic factors involved in NUS production.
- The robustness and simplicity of methods differ. Future research should consider using data with a finer resolution to improve the accuracy of mapping. This will help enhance the delineation of land suitability in marginalised agricultural communities that are known to be highly heterogeneous. The application of sensors mounted on un-manned aerial vehicles can validate satellite-derived data and capture high-resolution images [145,146]. The use of data derived from blockchain, cloud computing, big data and IoT technologies can improve the reliability and relevance of land suitability, especially in areas with high ecological risk.
- Future studies should focus on using new predictive tools in forecasting. It is observed that the majority of the studies in resource allocation utilised primitive GIS techniques. Future studies should focus on combining the GEPIC model with other methods to assess spatial distribution and stimulate the production of crops. The GEPIC model is used for predicting crop production levels incorporating near-real-time changes in the crop environment which can be integrated with other techniques for improved decision-making.

## 6 Conclusions

The review used a scoping method to acquire and synthesise information on land suitability for crop species. Robust land suitability methods are essential to developing land suitability maps to improve current and future planning on crop production guidelines, climate change issues and environmental management. The FAO land evaluation framework is the methods and provides the guidelines to delineate crop suitability maps. Modern land suitability methods are gaining popularity in cropland suitability analysis. The commonly used MCDM methods are AHP and fuzzy. The use of current and future climate change projections in LSA is the way forward for sustainable agriculture and food security. Qualitative and quantitative approaches must be integrated into a unique hybrid land evaluation system to improve the land evaluation approach. The review is expected to improve NUS land evaluation and provide researchers and decision-makers with most robust methods in developing LSA for NUS.

## Supplementary Material

Supplementary material

## Figures and Tables

**Table 1:** Description of hybrid land evaluation systems used in cropland suitability assessments.

Author	Methods	Crops
Bagherzadeh and Gholizadeh [40]	ANN, TOPSIS	Alfalfa
Bagherzadeh et al. [41]	ANN, Fuzzy	Soybean
Danvi et al. [42]	ML_BL	Rice
Deng et al. [43]	AHP, Fuzzy	Alfalfa
Estes et al. [44]	MaxEnt, GAM, DSSAT	Maize
Jiao and Liu [45]	ANN, GA	Rice
Manna et al. [46]	MicroLEIS, WAP, CropSyst	Maize
Pilehforooshha et al. [47]	CA, Fuzzy, GP	Multiple crops
van Lanen et al. [48]	ALES, WOFOST	Multiple crops
Jafarzadeh et al. [49]	Simple Limitation Method (SLM), Limitation Method regarding Number and Intensity (LMNI) square root and storie	Maize, Potato, alfalfa, onion
Habibie et al. [37]	ML, AHP	Maize
Lopez-Blanco et al. [50]	ML, GAEZ	Maize
Raza et al. [51]	ML, AHP	Rice
Seyedmohammadi et al. [52]	SAW, TOPSIS, Fuzzy	maize, rapeseed, soybean

**Table 2:** Description of traditional methods used in land suitability assessments.

Methods	Crops	NUS Yes/No	Thematic Factors			
			Climate	Soil and Landscape	Socio-Economic	LULC
Parametric	Wheat	No		^2^ N-P-K, Zn, Tex, Dep, Topo, SS, HP, HC, WHC, EC, ESP, CaCO_4_, pH	No	No
Boolean Logic, Maximum Limiting factor	Rice	No	P, T, RH, Flooding	D, Dep, CEC, BSP, pH, OC	No	No
WLC	Rice and Soybean	No	P, T, LGP, Stream order, discharge	Tex, OC, Phosphorus, pH, Drain, S, H, S, Dep, fertility	Land tenure, roads, markets, credit systems, incentive benefits	Yes
WO	Rice	No	P, T, SR, PET, AWC	ESP, Tex, S, G, Silt, clay	Land reforms	Yes
Square root mean	Wheat,^1^ *Sorghum,* Alfalfa, Barley, Maize, Rice, *Cassava,* Groundnut	Yes	P, RH, T, SR,	Dep, Tex, OC, ST, S, CaSO_4_, EC, CEC, ESP, Drain	No	Yes
Expert Knowledge, FAO method	Chemoriya		P, T, LGP, RH	SG, Tex, Dep, CEC, OM	No	No
Qualitative approach	Maize, *Pearl millet, Foxtail* *millet,* Potato, Vegetable	Yes		S, As, SG, H	Income	Yes
GAEZ	Wheat, Maize, Rice, Soybean		Min and Max T, P, RH, vapour pressure	SG, H, S	No	Yes
Computer overlay	Canola, Soybean	No	P,T	As, H, S, Tex, pH, EC	No	No

1 The italicised crop is considered as a priority Neglected and Underutilised species within Africa.

2 List of abbreviations: Aridity index (AI), Aspect (As), Base Saturation Percentage (BSP), Boron Toxicity (BT), Bulk Density (DB), Calcium Carbonate Equivalent (CCE), Cation Exchange Capacity (CEC), Depth (D), Depth to Water-Table (DTW), Dry month/Length of the dry season (DM), Effective depth (Dep), Electrical Conductivity Or Salinity (EC), Elevation (H), Flood (F), Groundwater (GW), Growing degree days (GDD), Gypsum (% CaSO4), Hard Pan (HP), Hydraulic Conductivity (HC), Irrigation Water Use (IWU), Land use land cover (LULC), Length of growing period (LGP), Length of the phenological period (LPP), Nitrogen-Phosphorus-Potassium (N-P-K), Organic Carbon (OC), Post-harvest technology (PHT), Potential evapotranspiration (PET), Rainfall (P), Relative humidity (RH), Slope (S), Sodicity (ESP), Sodium Adsorption Ratio (SAR), Soil drainage (Drain), Soil erosion (SE), Soil Groups/Soil, Soil Moisture (SM), Soil pH (pH), Soil Type (ST), Solar Radiation (SR), Sunshine hours (SH), Surface Stoniness (SS), Temperature (T), Temperature degree day (TDD), Texture (Tex), Topography (Topo), Types (SG), Water Holding Capacity (WHC),Weighed Overlay (WO), Weighted Linear Combination (WLC), Wet month (WM), Zinc (Zn).

**Table 3:** A description of modern methods used in land suitability assessments. References to the showcased methods can be found in the Supplementary Materials.

Methods	Crops	NUS	Thematic Factors			
			Climate	Soil and Landscape	Socio-Economic	LULC
AHP	Maize, Potato, Saffron, Rice, Grapes, Wheat, Sugarcane,	No	^2^ P, PET, Max T, Min T, RH, GDD, frost, SH	N-P-K, Zn, D, Tex, Dep, Topo, SS, HP, HC, WHC, EC, ESP, CaCO_4_, pH, OM, sand dune waviness, SE, Drain, DWT, SG, S, As, H	Infrastructure, Population, Literacy, Labour force, distance to road, economics index	Yes
Fuzzy methods	^1^ *Cassava,* Groundnut, Maize, *Millet,* Rice, Soybean, *Sorghum,* Barley, Spinach, Wheat, Rye, Oats, Sugarbeet, Hybrid Poplar	Yes	P, T, LGP, Stream order, discharge	Tex, Phosphorus, pH, Drain, S, H, S, Dep, fertility, Dep, Ca, Mg, K, CEC, OC, pH, H, Water availability, Gravel, Cobbles, EC, ESP, WHC, Tex, pH, OM	Market land value per acre, roads	Yes
Use of crop models: GIS-based Environmental Policy Integrated Climate (EPIC) model, Almagra, ECOCROP, CROPWAT	Sweet Potato, *Sorghum*, Soybean, Wheat, Maize	Yes	P, T, LGP, RH, SR, WM, AWC, AET, LGP, PET	Dep, Tex, Drain, EC ESP, CEC, pH, OC, BD, OM	GDP, Population, Undernutrition data	No
Machine learning related methods: Artificial Neural Networks, TOPSIS, Bayesian Networks (BNs), Goal programming Species distribution models, for example, (e.g., MaxEnt)	Wheat, Barley, Maize, Alfalfa, Potato, Wheat, *Cassava*	Yes	P, T, AI, PET, frost days, Chill hours, SR, AEP	Tex, EC, ESP, CaCO_4_, Gravel, Dep, OC, pH, S, Drain, F, CaSO_4_, OC, Tex, Thickness of tilth, S, N-P-K, Water conservancy, SG	Income, population, roads	Yes

1 The italicised crop is considered as a priority Neglected and Underutilised species within Africa.

2 List of abbreviations: Aridity index (AI), Aspect (As), Base Saturation Percentage (BSP), Boron Toxicity (BT), Bulk Density (DB), Calcium Carbonate Equivalent (CCE), Cation Exchange Capacity (CEC), Depth (D), Depth to Water-Table (DTW), Dry month/Length of the dry season (DM), Effective depth (Dep), Electrical Conductivity Or Salinity (EC), Elevation (H), Flood (F), Groundwater (GW), Growing degree days (GDD), Gypsum (% CaSO4), Hard Pan (HP), Hydraulic Conductivity (HC), Irrigation Water Use (IWU), Land use land cover (LULC), Length of growing period (LGP), Length of the phenological period (LPP), Nitrogen-Phosphorus-Potassium (N-P-K), Organic Carbon (OC), Post-harvest technology (PHT), Potential evapotranspiration (PET), Rainfall (P), Relative humidity (RH), Slope (S), Sodicity (ESP), Sodium Adsorption Ratio (SAR), Soil drainage (Drain), Soil erosion (SE), Soil Groups/Soil, Soil Moisture (SM), Soil pH (pH), Soil Type (ST), Solar Radiation (SR), Sunshine hours (SH), Surface Stoniness (SS), Temperature (T), Temperature degree day (TDD), Texture (Tex), Topography (Topo), Types (SG), Water Holding Capacity (WHC),Weighed Overlay (WO), Weighted Linear Combination (WLC), Wet month (WM), Zinc (Zn).

## Data Availability

The datasets generated during and/or analysed during the current study are available from the corresponding author on reasonable request.
