# Supplementary material for: Evaluation of Land Suitability Methods with Reference to Neglected and Underutilised Crop Species: A Scoping Review
Source: Land (Basel). Author manuscript; Available in PMC 2024 Jul 19. (PMC7616268; doi:10.3390/land10020125)
Supplement: Supplementary material [file EMS197512-supplement-Supplementary_material.pdf]

Supplementary Materials

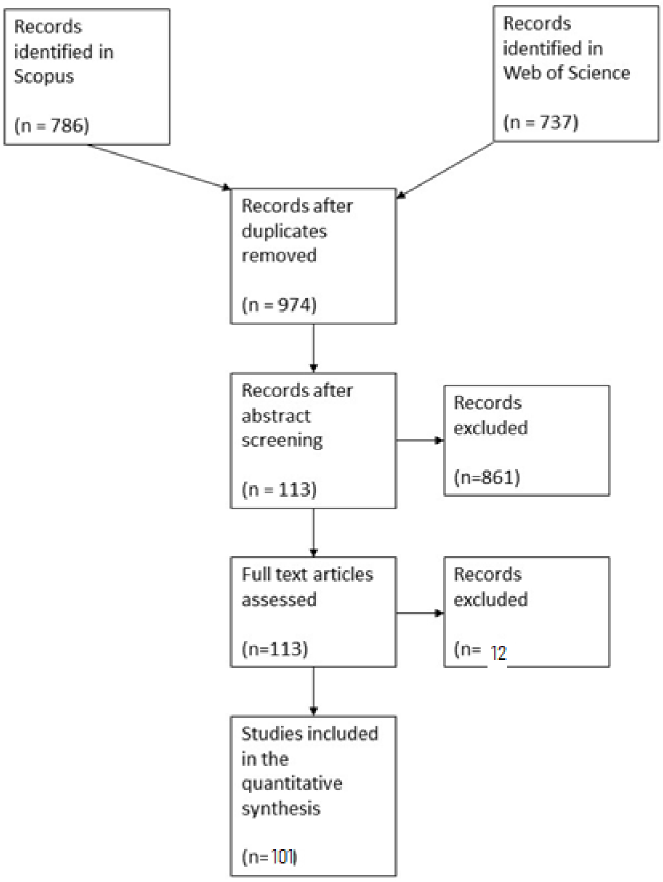

Figure S1. Preferred reporting items for systematic reviews and meta-analyses (PRISMA) flow chart diagram.

Table S1. Applicability and thematic factors used in traditional methods in crop suitability mapping.

AuthorsCountry		Objective of the Study	Methods Used or Model	Crop	NUS (Yes/No)	Thematic Factors			
	Country					Climatic	Soil and Landscape	Social-Economic Factors	LULC
[1]	Egypt	Spatial model for land suitability assessment	Parametric	Wheat	No		N-P-K, Zn, D, Tex, Dep, Topo, SS, HP, HC, WHC, EC, ESP, CaCO3, pH	No	No
[2]	Benin	Determine suitable areas for rice production	Boolean Logic, Maximum Limiting factor	Rice	No	P, T, RH, R, Flooding	D, Dep, CEC, BSP, pH, OC	No	No
[3]	Ghana	Most suitable areas for inland valley rice	WLC	Rice	No	P, LGP, Stream order, discharge	S, fertility, pH, N, OC, EC, CEC, BSP land reforms, Tex, ESP	Land tenure, roads, markets, credit systems, incentive benefits	Yes
[4]	Kenya	Evaluating the suitability of rice	WO	Rice	No	No	ESP, Tex	Land reforms	Yes
[5]	Ghana	Evaluating the suitability of rice	WLC	Rice	No	P, T, LGP, Stream order, discharge	Slope, Dep, fertility	Social-economic	No
[6]	Ethiopia	Land suitability analysis	Square root mean, WLC	Wheat, Sorghum	Yes	P, T	Dep, Tex, OC, D, Soil type, S	No	No
	Iran	Use of a model	Storie, Square root	Wheat, alfalfa, Barley, maize,	No	RH, T SR,	S, Tex, % CaSO4, EC, CEC, ESP, Drain	No	Yes
[7]	Zambia	Evaluation of land suitable for soybean	WLC	Soybean	No		Tex, OC, Phosphorus, pH, Drain, S, H	Roads	No
[8]	Senegal	Suitability analysis for rice	Storie, PCA	Rice, Cassava, Groundnut	No	P, T, RH, Wind, SH, PET	S, Drain, Coarse fragment, Dep, Tex, Clay, Silt, sand, CEC, OM, BSP, EC, ESP	No	No

[9]	Iran	Land suitability analysis	Computer overlay	Canola, Soybean	No	P, T	As, H, S, Tex, pH, EC	No	No
[10]	Spain	Land evaluation using biophysical factors	FAO, Statistics	Alfalfa, maize, rice, sunflower	No	LGP, SR, T, TWU, hailstorms, winds, Flood risk	Fertility, Dep, Tex, CEC, pH, BSP, OC	No	No
[11]	Burundi	Land evaluation	Sys	Wheat, Pea bean, maize, potato	No	P, LGP, T, RH, SH	S, Drain, H, Dep, SS, Tex, CEC, pH, BSP, OC	No	Yes
[12]		Crop specific suitability	Expert Knowledge, FAO method	Cherimoya	No	P, T, LGP, RH	SG, Tex, Dep, CEC, OM	No	No
[13]	China	Land suitability for sustainable development	Qualitative approach	Maize, Pearl millet, Foxtail millet, Potato, Apple, vegetable	Yes		S, As, SG, H	Income	Yes
[14]	Global	Spatial assessment of heat stress risk	GAEZ	Wheat, maize, rice, Soybean	No	Max and min T,	S, H	No	Yes
[15]	Africa	Impacts of climate change on agro-ecosystem	GAEZ	wheat, maize	No	Min and Max T, P, RH, vapour pressure	SG, H, S	No	Yes

Table S2. A list of analytic hierarchy process methods and factors used to delineate land suitability for crops.

Authors	Country	Objective of the Study	Method Used or Model	Crop	NUS (Yes/No)	Thematic Factors			
						Climatic	Soil and Landscape	Social-Economic Factors	LULC
[16]	Mexico	To map areas for maize and potatoes	AHP	Maize, Potatoes	No	P, PET, Max T, Min T,	Tex, Dep, S, H	No	Yes
[17]	Kenya	Rice suitability	AHP	Rice	No	T, RH	Tex, pH, Drain, S	No	No
[18]	Afghanistan	Use of logic scoring to improve AHP	AHP	Safron	No	P, T	SG, S, As, H	Road, economics index	Yes

[19]	Australia	To map potential growing areas for grapes	AHP-WLC	Grapes, pasture, Bluegum	No	P, TDD, Frost	S, As, Drain, PH, ESP, Dep, Tex, EC	No	No
[20]	India	Wheat suitability mapping	AHP-WO	Wheat	No		Tex, pH, ESP, EC, Drain, N-P-K, S, GW	No	Yes
[21]	Australia	Evaluating the uncertainty of power of AHP	AHP-Fuzzy	Ryegrass, Wheat	No	P, T	pH, WHC, Coarse fragment, Dep, Tex, EC, Drain	No	No
[22]	Brazil	To map areas that highly suitable for sugarcane	AHP	Sugarcane	No	P, T	SG, H	Infrastructure, Population, Literacy, Labor force	Yes
[23]	Iran	To assess the suitability of saffron	AHP-WO	Saffron	No	P, T, SH, Frost, RH	S, As, H, EC, pH, Tex	No	No
[13]	China	Sensitivity analysis for MCE	AHP-OAT-WLC	Wheat	No		Tex, Dep, OM, sand dune waviness, SE, Drain, DWT	No	No

List of abbreviations: Land use land cover (LULC), topography (Topo), surface stoniness (SS), hard pan (HP), hydraulic conductivity (HC), water holding capacity (WHC), groundwater (GW), soil moisture (SM), depth to water-table (DTW), temperature degree day (TDD), aridity index (AI), temperature (T), dry month/ length of the dry season (DM), wet month (WM), rainfall (P), soil drainage (Drain), texture (Tex), effective depth (Dep), cation exchange capacity (CEC), base saturation percentage (BSP), soil pH (pH), organic carbon (OC), slope (S), aspect (As), elevation (H), potential evapotranspiration (PET), solar radiation (SR), sunshine hours (SH), soil erosion (SE), length of the phenological period (LPP), gypsum (% $CaSO_4$), relative humidity (RH), boron toxicity (BT), soil type (ST), weighed overlay (WO) and weighted linear combination (WLC). Depth to water table (DWT), irrigation/irrigation water use (IWU), length of growing period (LGP), post-harvest technology (PHT), growing degree days (GDD), calcium carbonate equivalent (CCE), electrical conductivity or salinity (EC); sodium adsorption ratio (SAR), sodicity (ESP), surface stoniness/rockiness (SS), soil groups/soil types (SG).

Table S3. A list of fuzzy logic technique methods and common factors used to delineate land suitability for crops.

Authors	Country	Objective of the Study	Method Used or Model	Crop	NUS (Yes/No)	Thematic Factors			
				:		Climatic	Soil and Landscape	Social-Economic Factors	LULC
[24]	Global	Global land resources allocation	Fuzzy	16 Crops	Yes	P, T	Tex, Coarse fragments	No	No
[25]	India	To evaluate arable land on selected crops	Fuzzy	Finger millet, paddy, groundnut	Yes		Tex, Drain, Gravel, CEC, BSP, pH	No	No
[26]	Global	To evaluate the difference between topsoil properties for the dominant soil mapping units between two global soil datasets.	Fuzzy	Cassava, Groundnut, Maize, Millet, Oil-palm, Potatoes, Rapeseed, Rice, Rye, Sorghum, Soy, SugarcaneSunflower, Wheat	Yes	P, T	CaSO4, pH, BSP, OC, EC, ESP	No	No
[27]s	Ghana	Maize land Suitability evaluation	Fuzzy	Maize	No		OC, CEC, Drain, Clay, pH	No	No
[28]	Iran	Land suitability for irrigated sugar beet	Fuzzy	Sugar beet	No	T, LGP	H, SE, EC, ESP, OC	No	No
[29]	Switzerland	Evaluation of crop-specific climate suitability	Fuzzy	Maize	No	P, T, GDD, SR, AET, LPP	No	No	No
[30]	Australia	Evaluating procedures of land suitability evaluation in slope areas	Fuzzy	Barley, cotton, spinach, wheat, rye, maize, oats, sorghum	Yes		S, Drain, Gravel, Cobbles, EC, ESP, WHC, Tex, Dep, CEC, pH, OM	No	No

List of abbreviations: Land use land cover (LULC), topography (Topo), surface stoniness (SS), hard pan (HP), hydraulic conductivity (HC), water holding capacity (WHC), groundwater (GW), soil moisture (SM), depth to water-table (DTW), temperature degree day (TDD), aridity index (AI), temperature (T), dry month/ length of the dry season (DM), wet month (WM), rainfall (P), soil drainage (Drain), texture (Tex), effective depth (Dep), cation exchange capacity (CEC), base saturation percentage (BSP), soil pH (pH), organic carbon (OC), slope (S), aspect (As), elevation (H), potential evapotranspiration (PET), solar radiation (SR), sunshine hours

(SH), soil erosion (SE), length of the phenological period (LPP), gypsum (% $CaSO_4$), relative humidity (RH), boron toxicity (BT), soil type (ST), weighed overlay (WO) and weighted linear combination (WLC). Depth to water table (DWT), irrigation/irrigation water use (IWU), length of growing period (LGP), post-harvest technology (PHT), growing degree days (GDD), calcium carbonate equivalent (CCE), electrical conductivity or salinity (EC); sodium adsorption ratio (SAR), sodicity (ESP), surface stoniness/rockiness (SS), soil groups/soil types (SG).

Table S4. A list of crop models and factors used to delineate land suitability for crops.

Authors	Country	Objective of the Study	Method Used or Model	Crop	NUS (Yes/No)	Thematic Factors			
						Climatic	Soil and Landscape	Social-Economic Factors	LULC
[31]	United Kingdom	To delineate current and future land suitability for potato	Pedo-climatic functions, PSMD	Potato	No	P, T, AET, LGP	Dep, Tex, OM, S, SS,	No	No
[32]	Europe	Agro-climatic suitability	Water deficit Method	Maize	No	P, T, AWC		No	No
[33]	Africa	Assessment of current and future hotspots of food insecurity in SSA	GEPIC	Cassava, sorghum, wheat, maize	Yes	P, T, SR, WM	Dep, sand, silt, BD, PH, OC	GDP, population, Undernutrition data	No
[34]	Ethiopia	The impacts of climate change on land suitability for rain-fed crops	Almagra model, Sys	Sweet potato, Sorghum, soybean, wheat, maize	Yes	P, T, PET,	Dep, Tex, Drain, Ec, ESP, CEC, pH, OC	No	No
[35]	Global	To identify regions potentially suitable for crops	ECOCROP	Groundnut, soybean, sugarcane	Yes	P, T		No	No
[36]	Africa	Assessment of climate change on sorghum suitability	ECOCROP	Sorghum	Yes	P, T	No	No	N

List of abbreviations: Land use land cover (LULC), topography (Topo), surface stoniness (SS), hard pan (HP), hydraulic conductivity (HC), water holding capacity (WHC), groundwater (GW), soil moisture (SM), depth to water-table (DTW), temperature degree day (TDD), aridity index (AI), temperature (T), dry month/ length of the dry season (DM), wet month (WM), rainfall (P), soil drainage (Drain), texture (Tex), effective depth (Dep), cation exchange capacity (CEC), base saturation percentage (BSP), soil pH (pH), organic carbon (OC), slope (S), aspect (As), elevation (H), potential evapotranspiration (PET), solar radiation (SR), sunshine hours (SH), soil erosion (SE), length of the phenological period (LPP), gypsum (% $CaSO_4$), relative humidity (RH), boron toxicity (BT), soil type (ST), weighed overlay (WO) and weighted linear combination (WLC). Depth to water table (DWT), irrigation/irrigation water use (IWU), length of growing period (LGP), post-harvest technology

(PHT), growing degree days (GDD), calcium carbonate equivalent (CCE), electrical conductivity or salinity (EC); sodium adsorption ratio (SAR), sodicity (ESP), surface stoniness/rockiness (SS), soil groups/soil types (SG).

Table S5. A list of machine learning related methods and common factors used to delineate land suitability for crops.

Authors	Country	Objective of the study	Method used or Model	Crop	NUS (YES/NO)	Thematic factors			
						Climatic	Soil and landscape	Social-economic factors	LULC
[37]	Iran	Evaluation of land suitability of soybean in semi-arid regions	ANN, Fuzzy	Soybean	No	P, T, LGP	Tex, EC, ESP, CaCO3, Gravel, Dep, Oc, pH, S, Drain, Flood, CaSO4	No	No
[38]	Indonesia	Agricultural land suitability	ANN	Rice	No	P, T	DM, Drain, Tex, Dep, CEC, pH, N-P-K, ESP, S	No	No
[39]	China	Paddy rice land evaluation	Fuzzy Neural Network, GA	Rice	No		OC, Tex, Thickness of tilth, S, N-P-K, Water conservancy, pH	No	No
[40]	Nepal	bioclimatic conditions to assess the suitability	Global Environmental Stratification Strata, (GenS), ecological niche modelling, Fuzzy	Banana, Coffee	No	P, T. AI, PET,	As S	No	Yes
[41]	South Africa	To compare suitability and productivity of maize	MaxEnt, GAM, DSSAT	Maize	No	P, T, SR, H	SG	No	Yes
[42]	Global	To evaluate potential areas for coffee	MaxEnt	Coffee	No	P, T, Diurnal T	No	No	No
[43]	Indonesia	Quantify the potential CO2 emissions reductions	Logistic Regression	Palm oil	No	P, T	H, S, Dep, Drain, pH, OC	Roads	Yes
[44]	Thailand	Use of MaxEnt to map suitability areas for cassava	MaxEnt	Cassava	Yes	SR, PET	H, SG, S, As	No	Yes

[45]	Thailand	Understand factors affecting the suitability of crops	MaxEnt	Cassava, rice	Yes		H, SG	Population, roads	Yes
[46]	Australia	Assessment of soil and enterprise	Regression tree	Potato, Hazelnuts	No	P, T, frost days, Chill hours	Dep, pH, EC, Clay, Drain, SS	No	No
[47]	Iran	Suitable cultivable lands and water resources to optimize potential areas for crop production	Goal programming	Wheat, alfalfa, potato, maize	No	P, T	SG, course fragments, EC, pH, CaCO3, GW, water bodies	No	Yes
[29]	Switzerland	Evaluating crop-specific climate suitable	Knowledge-based determination of factor suitabilities, rule-based approach, WLC, genetic algorithm (GA)			P, T	H,	No	No
[48]	Africa	To evaluate cocoa-growing regions of Ghana and Côte d'Ivoire	MaxEnt	Cocoa	No	P, T, AEP	No	No	No
	Iran	land allocation	Cellular automata (CA), Markov chain, fuzzy rule-based systems, goal programming, WLC method	Wheat, Barley, Maize, alfalfa, Potato, Wheat	No	P, T, RH	S, Dep, Tex, Flood, Drain	Income	No
[49]	China	Evaluate land suitable for wheat	ANN	Wheat	No	P, T, SH	N, OC		

List of abbreviations: Land use land cover (LULC), topography (Topo), surface stoniness (SS), hard pan (HP), hydraulic conductivity (HC), water holding capacity (WHC), groundwater (GW), soil moisture (SM), depth to water-table (DTW), temperature degree day (TDD), aridity index (AI), temperature (T), dry month/ length of the dry season (DM), wet month (WM), rainfall (P), soil drainage (Drain), texture (Tex), effective depth (Dep), cation exchange capacity (CEC), base saturation percentage (BSP), soil pH (pH), organic carbon (OC), slope (S), aspect (As), elevation (H), potential evapotranspiration (PET), solar radiation (SR), sunshine hours

(SH), soil erosion (SE), length of the phenological period (LPP), gypsum (% $CaSO_4$), relative humidity (RH), boron toxicity (BT), soil type (ST), weighed overlay (WO) and weighted linear combination (WLC). Depth to water table (DWT), irrigation/irrigation water use (IWU), length of growing period (LGP), post-harvest technology (PHT), growing degree days (GDD), calcium carbonate equivalent (CCE), electrical conductivity or salinity (EC); sodium adsorption ratio (SAR), sodicity (ESP), surface stoniness/rockiness (SS), soil groups/soil types (SG).

Heuristic Models

Heuristic-based models are approaches used in problem-solving, learning, or discovery that employ a practical method. They are not guaranteed to be optimal, perfect, logical, or rational, but instead are sufficient for reaching an immediate goal [50]. Heuristic methods can be used to speed up the process of finding a satisfactory solution and works with presence-only data such as BioClimatic, ANUCLAM, DOMAIN, FEM and HABITAT [51,52]. Such models can be used in NUS where production information is limited. The models are simple to use, but they tend to over-predict [51]. They require ground-truthing or use of crop simulation models to validate the suitability maps [45]. This type of model can operate with a small number of records but can not make quantitative predictions or provide confidence levels [53,54].

(a) Decision tree models

Decision tree methods are used for data mining and aid in classifying systems based on multiple covariates or for developing prediction algorithms for a target variable [55]. The algorithm is non-parametric and can accommodate large, complicated datasets without imposing a complicated parametric structure (REF). The model requires one to know machine learning and programming skills. Frequently used algorithms include ACE, S-Plus algorithms CART, C4.5, CHAID and QUEST [56]. They can be linked with GIS and remote sensing data, SPSS and SAS programs that can be used to visualize tree structure [55].

(b) Genetic algorithms

Artificial neural networks (ANNs) are among the most advanced methods in land suitability analysis, a non-linear mapping structure works with presence or absence data [57]. The model requires a high number of records and can be challenging to use in areas where data is minimal especially in the study of NUS. The models result tend to be general because it depends on the sample frame. The more the number of training data the better the suitability index such as Genetic Algorithm for Rule-Set Prediction (GARP), MaxEnt [58,59]. It is a general-purpose machine learning method with a simple and precise mathematical formulation for modelling species geographic distributions with presence data only [60]. The assumptions not always evident, some models like Maximum entropy niche-based modelling are often used for environmental studies but the principle of maximum entropy can be used to delineate areas suitable for crops (MaxEnt) (Fourcade et al., 2014; Phillips et al., 2009).

(c) Additive statistical models

The generalised linear models (GLM) and generalised additive models (GAM) are additive statistical models, sometimes called ecological niche models [61,62]. Generalised linear models are probably the most commonly used statistical methods in bioclimatic modelling and have proven their ability to predict NUS distribution [45]. The models require lots of reliable records and knowledge of the ecology [63,64].

Considering the limitations of GLM in capturing complex response curves, application of generalised additive models is being proposed for species suitability mapping (Secondi, 2014). The generalised additive model blends the properties of the generalised linear models and additive models. Generalised additive models are based on nonparametric regression, and unlike GLM, which does not impose the assumption that the data supports a particular functional form (normally linear) [65]. Here the response variable is the additive combination of the functions of the

independent variable. However, transparency and interpretability are compromised to accommodate this greater flexibility. Applications of GLM in NUS land suitability might of less use because NUS are usually grown by smallholder farmers. Therefore, to capture the heterogeneous landscape and dynamic social-economic factors, we need generalised additive models which accommodate nonparametric factors used to delineate NUS, only 29% used LULC (Table 2).

Table S6. Climatic and hydrology factors, soil and landscape, social and economic factors and land use land cover (The results are presented as percentage N = 64).

**Factor**	**AHP**	**CSM**	**Fuzzy**	**MLM**	**TM**	**Total**
Climate						
Temperature	16	9	9	17	20	71
Precipitation	14	11	8	20	14	67
Relative Humidity	8	-	-	2	8	18
Length of the growing period	-	3	2	3	6	14
Growing degree days	-	-	5	3	-	8
PET	-	2	-	2	5	9
SH	3	-	-	2	-	5
SR	-	-	2	2	2	6
AET	-	2	2	-	-	4
Frost	2	-	-	2	-	4
Hydrology						
Flood	-	-	-	-	3	3
AWC	-	2	-	-	2	4
Hail Storms	-	-	-	-	2	2
TDD	2	-	-	-	-	2
Winds	-	-	-	-	2	2
Chill hours	-	-	-	2	-	2
Stream order	-	-	-	-	2	2
Discharge	-	-	-	-	2	2
Drain	-	2	-	-	-	2
Soil and landscape						
Factors	AHP	CSM	Fuzzy	MLM	TM	Total
Texture	9	3	8	8	19	47
pH	8	3	8	8	13	40
Slope	5	3	3	11	14	36
Soil depth	6	5	5	8	11	35
EC	5	-	9	6	6	26
OC	-	3	8	6	9	26
CEC	-	-	9	5	11	25
Elevation	3	-	3	8	8	22
ESP	3	-	5	5	5	18
As	5	-	-	3	3	11
N-P-K	3	-	-	3	2	8
Gravel	2	-	2	3	2	9
OM	5	2	-	-	-	7
CaSO4	-	-	2	3	2	7
BSP	-	-	2	-	5	7
Clay	2	-	2	2	2	8
SG	-	-	-	3	3	6

Mg	2	-	3	-	-	5
Ca	2	-	2	-	-	4
Cl	2	-	-	2	-	4
Sand	2	-	-	-	2	4
Silt	-	2	-	-	2	4
Thickness of tilth	-	-	-	2	2	4
Phosphorus	-	-	-	-	2	2
DM	-	-	2	-	-	2
BD	-	2	-	-	-	2
Fertility	-	-	-	-	2	2
Cobbles	-	-	2	-	-	2
Zn	-	-	-	-	2	2
Social-economic factors						
Factor	AHP	CSM	Fuzzy	MLM	TM	Total
Road	3	-	2	2	3	10
Population	2	-	-	2	-	4
Income	-	-	-	2	2	4
labour force	3	-	-	-	-	3
Infrastructure	2	-	-	-	-	2
Literacy	2	-	-	-	-	2
Land tenure	-	-	-	-	2	2
Markets	-	-	-	-	2	2
Credit systems	-	-	-	-	2	2
Incentive benefits	-	-	-	-	2	2
Economics index	2	-	-	-	-	2
Land value	-	-	2	-	-	2
Undernutrition data	-	2	-	-	-	2
GDP	-	2	-	-	-	2
Distance to city	2	-	-	-	-	2
Land use land cover						
Factor	AHP	CSM	Fuzzy	MLM	TM	Total
LULC	6	-	3	9	11	29

List of abbreviations: Land use land cover (LULC), topography (Topo), surface stoniness (SS), hard pan (HP), hydraulic conductivity (HC), water holding capacity (WHC), groundwater (GW), soil moisture (SM), depth to water-table (DTW), temperature degree day (TDD), aridity index (AI), temperature (T), dry month/ length of the dry season (DM), wet month (WM), rainfall (P), soil drainage (Drain), texture (Tex), effective depth (Dep), cation exchange capacity (CEC), base saturation percentage (BSP), soil pH (pH), organic carbon (OC), slope (S), aspect (As), elevation (H), potential evapotranspiration (PET), solar radiation (SR), sunshine hours (SH), soil erosion (SE), length of the phenological period (LPP), gypsum (% $CaSO_4$), relative humidity (RH), boron toxicity (BT), soil type (ST), weighed overlay (WO) and weighted linear combination (WLC). Depth to water table (DWT), irrigation/irrigation water use (IWU), length of growing period (LGP), post-harvest technology (PHT), growing degree days (GDD), calcium carbonate equivalent (CCE), electrical conductivity or salinity (EC); sodium adsorption ratio (SAR), sodicity (ESP), surface stoniness/rockiness (SS), soil groups/soil types (SG).

References

1. El Baroudy, A. Mapping and evaluating land suitability using a GIS-based model. *Catena* **2016**, *140*, 96–104, doi:10.1016/j.catena.2015.12.010.
2. Danvi, A.; Jütten, T.; Giertz, S.; Zwart, S.J.; Diekkrüger, B. A spatially explicit approach to assess the suitability for rice cultivation in an inland valley in central Benin. *Agric. Water Manag.* **2016**, *177*, 95–106, doi:10.1016/j.agwat.2016.07.003.
3. Masoud, J.; Agyare, W.A.; Forkuor, G.; Namara, R.; Ofori, E. Modeling inland valley suitability for rice cultivation. *ARPN J. Eng. Appl. Sci.* **2013**, *8*, 9–19

4. Kuria, D.; Ngari, D.; Waithaka, E. Using geographic information systems (GIS) to determine land suitability for rice crop growing in the Tana delta. *J. Geogr. Reg. Plan.* **2011**, *4*, 525–532.
5. Thenkabail, P.S.; Fujii, H.; Gumma, M.K.; Namara, R.E. Spatial models for selecting the most suitable areas of rice cultivation in the Inland Valley Wetlands of Ghana using remote sensing and geographic information systems. *J. Appl. Remote. Sens.* **2009**, *3*, 033537, doi:10.1117/1.3182847.
6. Motuma, M.; Suryabhagavan, K.; Balakrishnan, M. Land suitability analysis for wheat and sorghum crops in Wogdie District, South Wollo, Ethiopia, using geospatial tools. *Appl. Geomatics* **2016**, *8*, 57–66, doi:10.1007/s12518-016-0168-5.
7. Munene, P.; Chabala, L.M.; Mweetwa, A.M. Land Suitability Assessment for Soybean (Glycine max (L.) Merr.) Production in Kabwe District, Central Zambia. *J. Agric. Sci.* **2017**, *9*, 74, doi:10.5539/jas.v9n3p74.
8. Diallo, M.-D.; Wood, S.A.; Diallo, A.; Mahatma-Saleh, M.; Ndiaye, O.; Tine, A.K.; Ngamb, T.; Guisse, M.; Seck, S.; Diop, A.; et al. Soil suitability for the production of rice, groundnut, and cassava in the peri-urban Niayes zone, Senegal. *Soil Tillage Res.* **2016**, *155*, 412–420, doi:10.1016/j.still.2015.09.009.
9. Kamkar, B.; Dorri, M.A.; Da Silva, J.A.T. Assessment of land suitability and the possibility and performance of a canola (Brassica napus L.)—soybean (Glycine max L.) rotation in four basins of Golestan province, Iran. *Egypt. J. Remote. Sens. Space Sci.* **2014**, *17*, 95–104, doi:10.1016/j.ejrs.2013.12.001.
10. Casasnovas, J.A.M.; Klaasse, A.; Nogués, J.; Ramos, M. Comparison between land suitability and actual crop distribution in an irrigation district of the Ebro valley (Spain). *Span. J. Agric. Res.* **2008**, *6*, 700, doi:10.5424/sjar/2008064-363.
11. Hennebert, P.; Tessens, E.; Tourenne, D.; Delvaux, B. Validation of a FAO land evaluation method by comparison of observed and predicted yields of five food crops in Burundi. *Soil Use Manag.* **1996**, *12*, 134–142, doi:10.1111/j.1475-2743.1996.tb00534.x.
12. Bydekerke, L.; Van Ranst, E.; Vanmechelen, L.; Groenemans, R. Land suitability assessment for cherimoya in southern Ecuador using expert knowledge and GIS. *Agric. Ecosyst. Environ.* **1998**, *69*, 89–98, doi:10.1016/s0167-8809(98)00090-5.
13. Chen, Y.; Yu, J.; Khan, S. The spatial framework for weight sensitivity analysis in AHP-based multi-criteria decision making. *Environ. Model. Softw.* **2013**, *48*, 129–140, doi:10.1016/j.envsoft.2013.06.010.
14. Teixeira, E.I.; Fischer, G.; Van Velthuizen, H.; Walter, C.; Ewert, F. Global hot-spots of heat stress on agricultural crops due to climate change. *Agric. For. Meteorol.* **2013**, *170*, 206–215, doi:10.1016/j.agrformet.2011.09.002.
15. Fischer, G.; Shah, M.; Tubiello, F.N.; Van Velhuizen, H. Socio-economic and climate change impacts on agriculture: An integrated assessment, 1990–2080. *Philos. Trans. R. Soc. B Biol. Sci.* **2005**, *360*, 2067–2083, doi:10.1098/rstb.2005.1744.
16. Ceballos-Silva, A.; López-Blanco, J. Delineation of suitable areas for crops using a Multi-Criteria Evaluation approach and land use/cover mapping: A case study in Central Mexico. *Agric. Syst.* **2003**, *77*, 117–136, doi:10.1016/s0308-521x(02)00103-8.
17. Kihoro, J.; Bosco, N.J.; Murage, H. Suitability analysis for rice growing sites using a multicriteria evaluation and GIS approach in great Mwea region, Kenya. *SpringerPlus* **2013**, *2*, 265, doi:10.1186/2193-1801-2-265.
18. Wali, E.; Datta, A.; Shrestha, R.P.; Shrestha, S. Development of a land suitability model for saffron (Crocus sativus L.) cultivation in Khost Province of Afghanistan using GIS and AHP techniques. *Arch. Agron. Soil Sci.* **2015**, *62*, 1–14, doi:10.1080/03650340.2015.1101519.
19. Hood, A.; Cechet, B.; Hossain, H.; Sheffield, K. Options for Victorian agriculture in a "new" climate: Pilot study linking climate change and land suitability modelling. *Environ. Model. Softw.* **2006**, *21*, 1280–1289, doi:10.1016/j.envsoft.2005.04.022.
20. Dadhich, G.; Patel, P.R.; Kalubarme, M.H. Agriculture land suitability evaluation for wheat cultivation using geomatics for Patan District, India. *Int. J. Agric. Resour. Gov. Ecol.* **2017**, *13*, 91, doi:10.1504/IJARGE.2017.084038.
21. Benke, K.; Pelizaro, C. A spatial-statistical approach to the visualisation of uncertainty in land suitability analysis. *J. Spat. Sci.* **2010**, *55*, 257–272, doi:10.1080/14498596.2010.521975.
22. De Alkimim, A.F.; Sparovek, G.; Clarke, K.C. Converting Brazil's pastures to cropland: An alternative way to meet sugarcane demand and to spare forestlands. *Appl. Geogr.* **2015**, *62*, 75–84, doi:10.1016/j.apgeog.2015.04.008.
23. Maleki, F.; Kazemi, H.; Siahmarguee, A.; Kamkar, B. Development of a land use suitability model for saffron (Crocus sativus L.) cultivation by multi-criteria evaluation and spatial analysis. *Ecol. Eng.* **2017**, *106*, 140–153, doi:10.1016/j.ecoleng.2017.05.050.
24. Zabel, F.; Putzenlechner, B.; Mauser, W. Global Agricultural Land Resources—A High Resolution Suitability Evaluation and Its Perspectives until 2100 under Climate Change Conditions. *PLoS ONE* **2014**, *9*, e107522, doi:10.1371/journal.pone.0107522.
25. Ahamed, T.N.; Rao, K.G.; Murthy, J. GIS-based fuzzy membership model for crop-land suitability analysis. *Agric. Syst.* **2000**, *63*, 75–95, doi:10.1016/s0308-521x(99)00036-0.

26. Avellan, T.; Zabel, F.; Putzenlechner, B.; Mauser, W. A Comparison of Using Dominant Soil and Weighted Average of the Component Soils in Determining Global Crop Growth Suitability. *Environ. Pollut.* **2013**, *2*, 40, doi:10.5539/ep.v2n3p40.
27. Vlek, P.L.G.; Braimoh, A.K.; Stein, A. Land Evaluation for Maize Based on Fuzzy Set and Interpolation. *Environ. Manag.* **2004**, *33*, 226–238, doi:10.1007/s00267-003-0171-6.
28. Bagherzadeh, A.; Gholizadeh, A. Assessment of Soil Fertility for Sugar Beet Production Using Fuzzy AHP Approach and GIS in the Northeastern region of Iran. *Agric. Res.* **2018**, *7*, 61–71, doi:10.1007/s40003-018-0295-9.
29. Holzkamper, A.; Calanca, P.; Fuhrer, J. Identifying climatic limitations to grain maize yield potentials using a suitability evaluation approach. *Agric. For. Meteorol.* **2013**, *168*, 149–159, doi:10.1016/j.agrformet.2012.09.004.
30. Baja, S.; Chapman, D.M.; Dragovich, D. A conceptual model for defining and assessing land management units using a fuzzy modeling approach in GIS environment. *Environ. Manag.* **2002**, *29*, 647–661, doi:10.1007/s00267-001-0053-8.
31. Daccache, A.; Weatherhead, E.K.; Stalham, M.; Knox, J.W. Impacts of climate change on irrigated potato production in a humid climate. *Agric. For. Meteorol.* **2011**, *151*, 1641–1653, doi:10.1016/j.agrformet.2011.06.018.
32. Wolf, J.; Van Diepen, C. Effects of climate change on silage maize production potential in the European community. *Agric. For. Meteorol.* **1994**, *71*, 33–60, doi:10.1016/0168-1923(94)90099-x.
33. Liu, J.; Zehnder, A.J.B.; Yang, H. Drops for crops: Modelling crop water productivity on a global scale. *Glob. Nest J.* **2008**, *10*, 295–300.
34. Kibret, R.N.L.A.K. Climate Change Impact on Land Suitability for Rainfed Crop Production in Lake Haramaya Watershed, Eastern Ethiopia. *J. Earth Sci. Clim. Chang.* **2016**, *7*, 07, doi:10.4172/2157-7617.1000343.
35. Lane, A.; Jarvis, A. Changes in climate will modify the geography of crop suitability: Agricultural biodiversity can help with adaptation. *SAT eJournal* **2007**, *4* doi:10.3914/ICRISAT.0094.
36. Ramirez-Villegas, J.; Jarvis, A.; Läderach, P. Empirical approaches for assessing impacts of climate change on agriculture: The EcoCrop model and a case study with grain sorghum. *Agric. For. Meteorol.* **2013**, *170*, 67–78, doi:10.1016/j.agrformet.2011.09.005.
37. Bagherzadeh, A.; Ghadiri, E.; Darban, A.R.S.; Gholizadeh, A. Land suitability modeling by parametric-based neural networks and fuzzy methods for soybean production in a semi-arid region. *Model. Earth Syst. Environ.* **2016**, *2*, doi:10.1007/s40808-016-0152-4.
38. Wang, F. The Use of Artificial Neural Networks in a Geographical Information System for Agricultural Land-Suitability Assessment. *Environ. Plan. A: Econ. Space* **1994**, *26*, 265–284, doi:10.1068/a260265.
39. Jiao, L.; Liu, Y. Model of land suitability evaluation based on computational intelligence. *Geo-spatial Inf. Sci.* **2007**, *10*, 151–156, doi:10.1007/s11806-007-0053-9.
40. Ranjitkar, S.; Sujakhu, N.M.; Merz, J.; Kindt, R.; Xu, J.; Matin, M.A.; Ali, M.; Zomer, R.J. Suitability Analysis and Projected Climate Change Impact on Banana and Coffee Production Zones in Nepal. *PLoS ONE* **2016**, *11*, e0163916, doi:10.1371/journal.pone.0163916.
41. Estes, L.; Bradley, B.A.; Beukes, H.; Hole, D.G.; Lau, M.; Oppenheimer, M.G.; Schulze, R.; Tadross, M.; Turner, W.R. Comparing mechanistic and empirical model projections of crop suitability and productivity: Implications for ecological forecasting. *Glob. Ecol. Biogeogr.* **2013**, *22*, 1007–1018, doi:10.1111/geb.12034.
42. Ovalle-Rivera, O.; Läderach, P.; Bunn, C.; Obersteiner, M.; Schroth, G. Projected Shifts in Coffea arabica Suitability among Major Global Producing Regions Due to Climate Change. *PLoS ONE* **2015**, *10*, e0124155, doi:10.1371/journal.pone.0124155.
43. Austin, K.G.; Kasibhatla, P.S.; Urban, D.L.; Stolle, F.; Vincent, J. Reconciling Oil Palm Expansion and Climate Change Mitigation in Kalimantan, Indonesia. *PLoS ONE* **2015**, *10*, e0127963, doi:10.1371/journal.pone.0127963.
44. Heumann, B.W.; Walsh, S.J.; McDaniel, P.M. Assessing the application of a geographic presence-only model for land suitability mapping. *Ecol. Informatics* **2011**, *6*, 257–269, doi:10.1016/j.ecoinf.2011.04.004.
45. Heumann, B.W.; Walsh, S.J.; Verdery, A.M.; McDaniel, P.M.; Rindfuss, R.R. Land Suitability Modeling Using a Geographic Socio-Environmental Niche-Based Approach: A Case Study from Northeastern Thailand. *Ann. Assoc. Am. Geogr.* **2013**, *103*, 764–784, doi:10.1080/00045608.2012.702479.
46. Kidd, D.; Webb, M.; Malone, B.; Minasny, B.; McBratney, A. Digital soil assessment of agricultural suitability, versatility and capital in Tasmania, Australia. *Geoderma Reg.* **2015**, *6*, 7–21, doi:10.1016/j.geodrs.2015.08.005.
47. Mockshell, J.; Kamanda, J. Beyond the agroecological and sustainable agricultural intensification debate: Is blended sustainability the way forward? *Int. J. Agric. Sustain.* **2018**, *16*, 127–149, doi:10.1080/14735903.2018.1448047.
48. Läderach, P.; Martinez-Valle, A.I.; Schroth, G.; Castro, N. Predicting the future climatic suitability for cocoa farming of the world's leading producer countries, Ghana and Côte d'Ivoire. *Clim. Chang.* **2013**, *119*, 841–854, doi:10.1007/s10584-013-0774-8.

49. Wang, D.-C.; Li, C.-J.; Song, X.; Wang, J.-H.; Yang, X.-D.; Huang, W.-J.; Wang, J.-Y.; Zhou, J.-H. Assessment of Land Suitability Potentials for Selecting Winter Wheat Cultivation Areas in Beijing, China, Using RS and GIS. *Agric. Sci. China* **2011**, *10*, 1419–1430, doi:10.1016/s1671-2927(11)60135-1.
50. Mustafa, A.A.; Singh, M.; Sahoo, R.N.; Ahmed, N.; Khanna, M.; Sarangi, A. Land Suitability Analysis for Different Crops: A Multi Criteria Decision Making Approach using Remote Sensing and GIS. *Water Technol.* **2011**, *3*, 61–84.
51. Booth, T.H.; Nix, H.A.; Busby, J.R.; Hutchinson, M.F. Bioclim: The first species distribution modelling package, its early applications and relevance to most current MaxEnt studies. *Divers. Distrib.* **2013**, *20*, doi:10.1111/ddi.12144
52. Duan, R.-Y.; Kong, X.-Q.; Huang, M.-Y.; Fan, W.-Y.; Wang, Z.-G. The Predictive Performance and Stability of Six Species Distribution Models. *PLoS ONE* **2014**, *9*, e112764, doi:10.1371/journal.pone.0112764.
53. Xu, T.; Hutchinson, M.F. New developments and applications in the ANUCLIM spatial climatic and bioclimatic modelling package. *Environ. Model. Softw.* **2013**, *40*, 267–279, doi:10.1016/j.envsoft.2012.10.003.
54. Xu, T.; Hutchinson, M.F. New developments and applications in the ANUCLIM spatial climatic and bioclimatic modelling package. In Proceedings of the 6th International Congress on Environmental Modelling and Software, Leipzig, Germany, 1–5 July 2012.
55. Pecchi, M.; Marchi, M.; Burton, V.; Giannetti, F.; Moriondo, M.; Bernetti, I.; Bindi, M.; Chirici, G. Species distribution modelling to support forest management. A literature review. *Ecol. Model.* **2019**, *411*, 108817, doi:10.1016/j.ecolmodel.2019.108817.
56. Kotsiantis, S. Decision trees: A recent overview. *Artif. Intell. Rev.* **2011**, *39*, 261–283, doi:10.1007/s10462-011-9272-4.
57. Basse, R.M.; Omrani, H.; Charif, O.; Gerber, P.; Bódis, K. Land use changes modelling using advanced methods: Cellular automata and artificial neural networks. The spatial and explicit representation of land cover dynamics at the cross-border region scale. *Appl. Geogr.* **2014**, *53*, 160–171, doi:10.1016/j.apgeog.2014.06.016.
58. Anderson, R.P.; Lew, D.; Peterson, A. Evaluating predictive models of species' distributions: Criteria for selecting optimal models. *Ecol. Model.* **2003**, *162*, 211–232, doi:10.1016/s0304-3800(02)00349-6.
59. Phillips, S.J.; Anderson, R.P.; Schapire, R.E. Maximum entropy modeling of species geographic distributions. *Ecol. Model.* **2006**, *190*, 231–259, doi:10.1016/j.ecolmodel.2005.03.026.
60. Sharma, R.; Kamble, S.S.; Gunasekaran, A. Big GIS analytics framework for agriculture supply chains: A literature review identifying the current trends and future perspectives. *Comput. Electron. Agric.* **2018**, *155*, 103–120, doi:10.1016/j.compag.2018.10.001.
61. Oppel, S.; Meirinho, A.; Ramírez, I.; Gardner, B.; O'Connell, A.F.; Miller, P.I.; Louzao, M. Comparison of five modelling techniques to predict the spatial distribution and abundance of seabirds. *Biol. Conserv.* **2012**, *156*, 94–104, doi:10.1016/j.biocon.2011.11.013.
62. Austin, M. Species distribution models and ecological theory: A critical assessment and some possible new approaches. *Ecol. Model.* **2007**, *200*, 1–19, doi:10.1016/j.ecolmodel.2006.07.005.
63. Peterson, A.T. Uses and Requirements of Ecological Niche Models and Related Distributional Models. *Biodivers. Informatics* **2006**, *3*, doi:10.17161/bi.v3i0.29.
64. Sillero, N. What does ecological modelling model? A proposed classification of ecological niche models based on their underlying methods. *Ecol. Modell.* **2011**, *222*, 1343–1346, doi:10.1016/j.ecolmodel.2011.01.018.
65. Warren, D.L. In defense of "niche modeling." *Trends Ecol. Evol.* **2012**, *27*, 497–500.
